# Parliament and the Profession

**Published:** 1917-10-27

**Authors:** 


### Parliament and the Profession.

Parliament was resumed on Tuesday, October 16, after the autumn recess.

### Women Workers in V.A.D. Hospitals.

Captain Bennet-Goldney, in a question to the Prime Minister, mentioned the services of women workers in V.A.D. hospitals, and asked if it might be possible to award them, as a mark of national gratitude, some honourable but inexpensive recognition such as a distinctive ribbon. Mr. Bonar Law, who replied, said that every endeavour is being made, in conjunction with the Chairman of the Joint War Committee of the British Red Cross Society and Order of St. John of Jerusalem and the Central Joint Voluntary Aid Detachment Committee, to afford such recognition as is possible to the service of those ladies, and when final details respecting the award of the Order of the British Empire have been settled further opportunities for award will be available. Neither the War Office nor the other departments concerned regard with favour the suggestion that a distinctive ribbon should be instituted.

### Ministry of Health.

Mr. Bonar Law informed Mr. Dillon that, although the question of a Ministry of Health has not reached any widely agreed solution, steps are being taken which will, it is hoped, secure substantial agreement amongst those who are actively engaged in the work of national health.

### Military Dental Officers.

Mr. Macpherson informed Mr. Pennefather that the total number of commissioned dental officers is 517.

### Expeditionary Force Medical Service.

Colonel Gretton asked if the Commission recently sent to examine into the Medical Service of the Expeditionary Force had reported; if not, when the Report might be expected, and if it would be laid upon the Table of the House. Mr. Macpherson stated that no Report had been received, nor could he say when it would be in the possession of the Army Council. No promise could be made in advance as to making the Report public.

### National Health Benefits and Air-Raids.

In reply to a question by Mr. Chancellor, Sir Edwin Cornwall stated that he is advised that approved societies would not be entitled to refuse sickness benefit to insured persons on the ground that their illness had been due to air-raids unless the injury arose out of the member's employment, in which case the matter would be one for compensation under the Workmen's Compensation Act.

### Royal Army Medical Corps.

Mr. Macpherson stated that the pay and allowances of a captain in the Territorial Force R.A.M.C. vary from 19s. to 23s. 2d. a day. The pay of a temporary lieutenant engaged on a contract basis is 24s. a day, inclusive. The captain is entitled to a larger gratuity on cessation and to other advantages, but the question of the comparative emoluments is under the consideration of the Council.

